# The quantum Ising model: finite sums and hyperbolic functions

**DOI:** 10.1038/srep15779

**Published:** 2015-10-30

**Authors:** Bogdan Damski

**Affiliations:** 1 Jagiellonian University, Institute of Physics, Łojasiewicza 11, 30-348 Kraków, Poland

## Abstract

We derive exact closed-form expressions for several sums leading to hyperbolic functions and discuss their applicability for studies of finite-size Ising spin chains. We show how they immediately lead to closed-form expressions for both fidelity susceptibility characterizing the quantum critical point and the coefficients of the counterdiabatic Hamiltonian enabling arbitrarily quick adiabatic driving of the system. Our results generalize and extend the sums presented in the popular Gradshteyn and Ryzhik Table of Integrals, Series, and Products.

In typical condensed matter setups the size of a system is thermodynamic and so the finite-size effects are negligible. The situation can be quite the opposite with quantum simulators, i.e., quantum systems such as cold atoms^1^,^2^ and ions^3^,^4^ that are tailored to emulate the challenging condensed matter models. One of the ultimate goals of this field is to emulate spin systems that are analytically and numerically unapproachable in the thermodynamic limit. Realization of such a goal requires substantial experimental progress, but it is reasonable to expect that on-demand emulation of arbitrary spin models is within experimental reach^5^. The current state-of-the-art experiments, however, manage to emulate spin systems composed of less than 20 spins^6^,^7^,^8^, where the finite-size effects come into play.

It is thus desirable to develop better understanding of finite-size effects in many-body spin systems to be able to (i) extrapolate the finite-size data to the thermodynamic limit; (ii) understand the limitations of the current generation of quantum emulators. Actually, the finite-size effects are interesting on their own in systems undergoing a quantum phase transition. Indeed, such systems are especially sensitive to the system size because their correlation length diverges around the critical point in the thermodynamic limit^9^,^10^. Additionally, we mention that finite-size systems are relevant for quantum engineering, where the goal is to prepare a system in a desired quantum state (see e.g. ref. 11).

Due to its exact solvability, the one dimensional quantum Ising model is a perfect system for analytical studies of a quantum phase transition^9^,^10^. Such studies have been extensively performed in the past in the thermodynamic limit. This limit allowed for the observation of singularities at the critical point and simplified the analytical computations^12^,^13^,^14^.

The calculations in the thermodynamic limit of the quantum Ising model rely on the replacement of sums with integrals. If we would be able to find closed-form expressions for such sums, we would get detailed analytical insights into the finite-size effects in a model system undergoing a quantum phase transition. The main goal of this work is to provide several closed-form expressions for the sums that appear in the studies of the quantum Ising model. Additionally, we briefly discuss two examples, where the use of our sums immediately leads to elegant expressions for both fidelity susceptibility capturing the key properties of the quantum critical point and the coefficients of the counterdiabatic Hamiltonian allowing for implementation of perfectly adiabatic dynamics on an arbitrarily short time scale.

## Results

There are four interestingly looking sums in Chapter 1.382 of ref. 15:


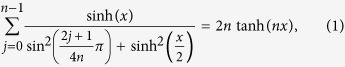



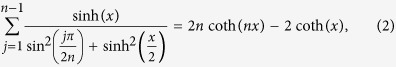



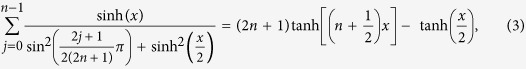



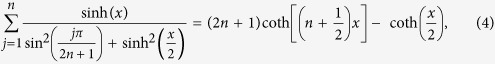


which are valid for integer *n* ≥ 1 (we assume such *n* everywhere in this article). To explain where these sums come from, we consider polynomials


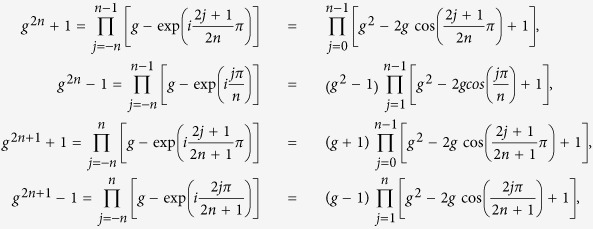


which can be also written as


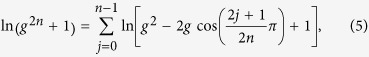



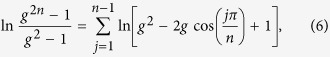



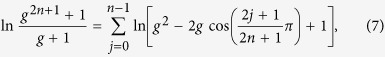



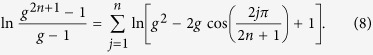


If we now substitute


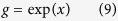


into Eqs (5, 6, 7, 8) and differentiate them with respect to *x*, we will get Eqs (1, 2, 3, 4) after simple algebra. As will be explained in the Discussion section, the parameter *g* (9) is the magnetic field in the quantum Ising model.

The sums (1)–(4) can be generalized in the following way


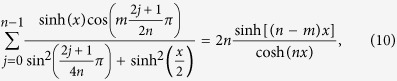



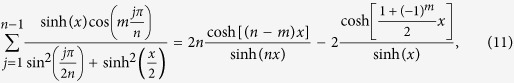



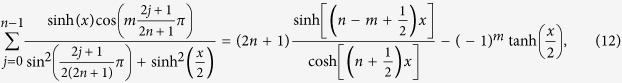



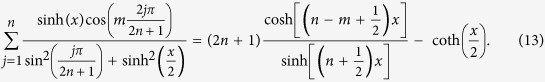


Eqs (10, 11) are valid for 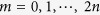, while Eqs (12-13) hold for 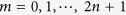. These results reproduce Eqs (1-4) for *m* = 0.

Additionally, we propose another set of identities


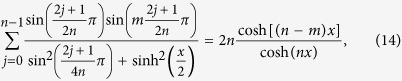



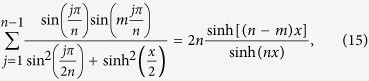



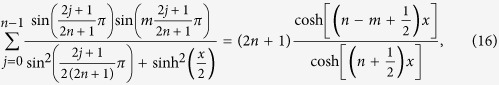



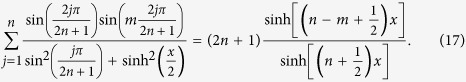


Eqs (14,15) yield a correct result for 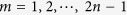, while Eqs (16,17) hold for 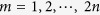.

The sums (10)–(17) have been conjectured by applying the techniques described in Appendix A of ref. 16. Two of them, (10) and (14), have been already proven in ref. 16. We will outline below the steps allowing for an inductive proof of all the sums. This way we will generalize the former results and simplify a bit the proof.

To compactify notation, we define two functions


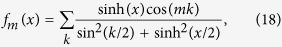



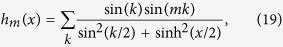


where the momenta *k* are chosen such that 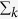 replaces 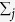 in Eqs (10-17). Next, we show that


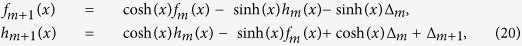


where 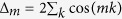. These recurrence relations can be derived by substituting


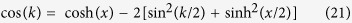


and


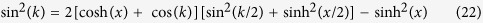


into  cos [(*m* + 1) *k*] = cos (*mk*) cos (*k*) - sin (*mk*) sin (*k*)  and  sin (*k*) sin[(*m* + 1) *k*] = sin (*k*) sin (*mk*) cos (*k*) + cos (*mk*) sin^2^ (*k*).

To proceed with the inductive proof, we find in an elementary way that


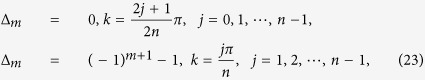


for 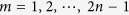 and


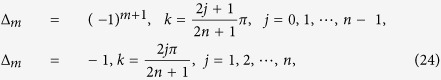


for 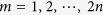.

First, we assume that 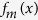 and 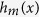 are given by the right-hand sides of Eqs( 10-13) and (14-17), respectively.

Second, we check that our inductive conjecture holds for *m* = 1. This is done by substituting Eqs (21,22) into Eqs (18,19) taken at *m* = 1 and then comparing the resulting expressions to Eqs (1, 2, 3, 4).

Third, it is a straightforward exercise to check that the expressions for 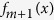 and 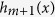 obtained from the recurrence equations (20) are exactly the same as the right-hand sides of the conjectured equations (10, 11, 12, 13, 14, 15, 16, 17) after the replacement of *m* by *m* *+* 1. The last remark is true provided that expressions for Δ_*m*_ are used in the recurrence equations for appropriate *m* listed below Eqs (23,24). This completes the proof that Eqs (10,11,14,15) are correct for 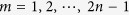, while Eqs (12,13,16,17) hold for 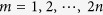. Additionally, one may substitute *m* = 2*n* into Eqs (10,11) and 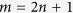 into Eqs (12,13) to immediately verify with Eqs (1, 2, 3, 4) that Eqs (10, 11, 12, 13) are correct for such *m* as well. This concludes the proof of Eqs (10, 11, 12, 13, 14, 15, 16, 17).

## Discussion

The results obtained in the previous section provide useful analytical tools for the studies of the quantum Ising model. The Hamiltonian of this model reads


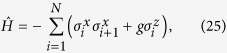


where *N* is the number of spins, 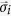 denote the Pauli matrices acting on the *i*-th spin, *g* is a magnetic field, and periodic boundary conditions are assumed 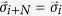. This exactly solvable model has many interesting properties^9^,^10^. We will mention two of them that are relevant for the present discussion.

First, the above Hamiltonian commutes with the so-called parity operator


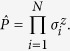


Therefore, its Hilbert space can be divided into positive and negative parity subspaces (eigenvalues of the parity operator are ±1 because 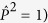. The parity of the ground state depends on both the system size and the direction of the magnetic field (see e.g. ref. 17).

Second, the quantum Ising model can be mapped to the non-interacting lattice fermionic model through the Jordan-Wigner transformation and then it can be diagonalized in the momentum space^9^,^12^,^18^. There are four possibilities for the choice of momenta *k* in a periodic system (see e.g. ref. 17). In the positive parity subspace of the even-sized system


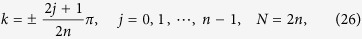


while in the negative parity subspace of such a system


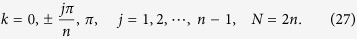


In the positive parity subspace of the odd-sized system


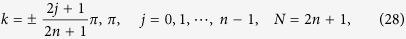


while in the negative parity subspace of such a system


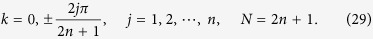


Forgetting about the irrelevant sign of *k* and the 0 and *π* momentum modes, the summations over these four choices of momenta correspond to the four summations over *j* seen in Eqs (10, 11, 12, 13, 14, 15, 16, 17).

What’s more, the denominator of all the sums that we have computed, 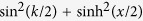, can be mapped to the form familiar from the Ising model computations,


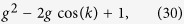


after setting


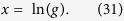


Note that we have already used this mapping at the beginning of the Results section (9). Since both the eigenvalues and eigenstates of quantum Ising model depend on the factor (30)—see e.g. ref. 9—it is not surprising that the sums involving it appear in the studies of this model.

Finally, we note that the sums involving positive integer powers of the factor (30) in the denominator can be easily generated through the differentiation of the sums (10)–(17) with respect to *x*. We are now ready to show two applications for the results of the previous section.

### Fidelity approach to quantum phase transitions

The location of the critical point and the critical exponent quantifying power-law divergence of the correlation length around the critical point can be extracted from the overlap between ground states corresponding to different external fields^19^,^20^,^21^,^22^,^23^,^24^. Such an overlap is called fidelity^19^. It defines the so-called fidelity susceptibility *χ* through a Taylor expansion in the field shift *δ*^25^


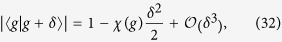


where 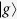 is a ground state of a Hamiltonian depending on an external field *g*. It can be easily shown that^19^


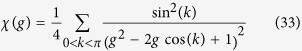


in the quantum Ising model (25); the restriction to 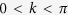 in the sum is applied to momenta from Eqs (26, 27, 28, 29).

Therefore, to compute the exact closed-form expression for fidelity susceptibility 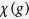 one needs to (i) determine the parity of the ground state and choose the appropriate momenta *k* from the list (26)–(29); (ii) choose the relevant sum from Eqs (14, 15, 16, 17), set *m* = 1 to obtain 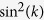 in the numerator of the summand, and compute derivative of the resulting expression with respect to *x* to square the summand’s denominator; (iii) substitute (31) into the resulting expression. One gets in the end


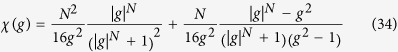


for all system sizes and magnetic fields. This expression was computed in a bit more complicated way in refs 17,26.

The quantum Ising model has the critical point at *g*_*c*_ = 1 separating the ferromagnetic phase (0 ≤ *g* < 1) from the paramagnetic phase (*g* > 1). It is thus expected that its ground states are most sensitive to the change of the magnetic field near the critical point. Therefore, fidelity (32) should have minimum near the critical point, and so fidelity susceptibility should have maximum there. One can use Eq. (34) to easily analytically compute the location of the maximum of fidelity susceptibility and quantify how fast it approaches *g*_*c*_ = 1 as the size of the system is increased. One can also study with the help of Eq. (34) the dependence of fidelity susceptibility on the system size and the distance from the critical point, which yields further insights into the quantum criticality of the Ising model (see ref. 26 for the details).

### Counterdiabatic driving

Perfectly adiabatic evolutions of a quantum system subjected to arbitrary time variation of an external field can be engineered by a proper modification of a Hamiltonian^11^,^27^,^28^,^29^.

For the quantum Ising chain this can be obtained after adding to the Hamiltonian (25)


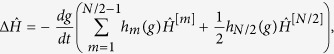


for even *N* and


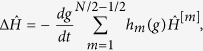


for odd *N*, where


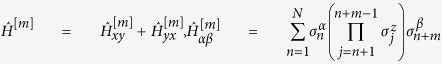


describes interactions of *m* + 1 adjacent spins. This so-called counterdiabatic Hamiltonian was computed for even-sized chains evolving in the positive parity subspace in ref. 30. We have checked that it can be written in such a form for systems of an arbitrary size evolving in a subspace of arbitrary parity.

It should be perhaps explained that the fairly complicated counterdiabatic Hamiltonian is obtained by “reverse-engineering”. It is written down first in the momentum space, where it takes a simple form, and then it is transformed to the real space form. It is thus assumed that the system evolves in the subspace of the Hilbert space of definite parity. Such an assumption can be made because parity is conserved during time evolutions with the counterdiabatic Hamiltonian: 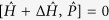.

The strength of the (*m* + 1)-body interactions in the counterdiabatic Hamiltonian is given by


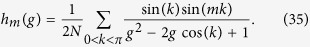


Using mapping (9) as well as Eqs (14, 15, 16, 17,26, 27, 28, 29), one immediately finds that for any system size *N* > 1 and 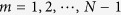



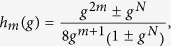


where the + (−) sign should be used for evolutions taking place in the positive (negative) parity subspaces of the Hilbert space. The closed-form expression for Eq. (35) in even-sized systems evolving in the positive parity subspace was previously reported in ref. 16.

Summarizing, we have derived several expressions for the sums leading to hyperbolic functions. They generalize and extend the results listed in the popular Gradshteyn and Ryzhik book^15^. We expect that they will be useful in different studies and illustrated their applicability to the quantum Ising model, which has provided the primary motivation for this work.

## Additional Information

**How to cite this article**: Damski, B. The quantum Ising model: finite sums and hyperbolic functions. *Sci. Rep.* **5**, 15779; doi: 10.1038/srep15779 (2015).
